# T-Lymphocyte Interactions with the Neurovascular Unit: Implications in Intracerebral Hemorrhage

**DOI:** 10.3390/cells11132011

**Published:** 2022-06-24

**Authors:** Samuel X. Shi, Samuel J. Vodovoz, Yuwen Xiu, Ning Liu, Yinghua Jiang, Prasad V. G. Katakam, Gregory Bix, Aaron S. Dumont, Xiaoying Wang

**Affiliations:** Clinical Neuroscience Research Center, Department of Neurosurgery & Neurology, Tulane University School of Medicine, New Orleans, LA 70112, USA; svodovoz@tulane.edu (S.J.V.); xiuyuwen@hotmail.com (Y.X.); nliu3@tulane.edu (N.L.); yjiang11@tulane.edu (Y.J.); pkatakam@tulane.edu (P.V.G.K.); gbix@tulane.edu (G.B.); adumont2@tulane.edu (A.S.D.); xwang51@tulane.edu (X.W.)

**Keywords:** intracerebral hemorrhage, T cells, neurovascular unit, immunology, stroke

## Abstract

In the pathophysiology of hemorrhagic stroke, the perturbation of the neurovascular unit (NVU), a functional group of the microvascular and brain intrinsic cellular components, is implicated in the progression of secondary injury and partially informs the ultimate patient outcome. Given the broad NVU functions in maintaining healthy brain homeostasis through its maintenance of nutrients and energy substrates, partitioning central and peripheral immune components, and expulsion of protein and metabolic waste, intracerebral hemorrhage (ICH)-induced dysregulation of the NVU directly contributes to numerous destructive processes in the post-stroke sequelae. In ICH, the damaged NVU precipitates the emergence and evolution of perihematomal edema as well as the breakdown of the blood–brain barrier structural coherence and function, which are critical facets during secondary ICH injury. As a gateway to the central nervous system, the NVU is among the first components to interact with the peripheral immune cells mobilized toward the injured brain. The release of signaling molecules and direct cellular contact between NVU cells and infiltrating leukocytes is a factor in the dysregulation of NVU functions and further adds to the acute neuroinflammatory environment of the ICH brain. Thus, the interactions between the NVU and immune cells, and their reverberating consequences, are an area of increasing research interest for understanding the complex pathophysiology of post-stroke injury. This review focuses on the interactions of T-lymphocytes, a major cell of the adaptive immunity with expansive effector function, with the NVU in the context of ICH. In cataloging the relevant clinical and experimental studies highlighting the synergistic actions of T-lymphocytes and the NVU in ICH injury, this review aimed to feature emergent knowledge of T cells in the hemorrhagic brain and their diverse involvement with the neurovascular unit in this disease.

## 1. Introduction

Intracerebral hemorrhage (ICH) is the deadliest subtype of stroke, accounting for 15–30% of global strokes per annum, and its prognosis remains devastating. ICH causes catastrophic brain damage and particularly poor prognoses in patients. In spite of improvements in control and prevention of risk factors, nationwide ICH incidence has increased in the past 15 years and minority populations continue to experience disparate ICH burden [1]. Higher rates of increase in ICH incidence among young and middle-aged Americans are particularly concerning and warrant continued research endeavors. In 2020 the global incidence of ICH was 3.41 million with substantial corresponding global deaths [1,2]. Despite this ongoing and growing public health burden, the standard approach to treatment remains restricted to symptomatic control [3,4]. Considering the urgent need for new interventional approaches for ICH, various large-scale multi-center randomized clinical trials (ISRCTN22153967, ChiCTR-TRC-12002026, NCT00784134, NCT01827046) investigating the efficacy of surgical interventional approaches for early hematoma removal have reported all no functional improvements in patients [5,6,7,8]. In light of the failure of hematomal evacuation interventions to demonstrate efficacy in improving patient prognosis, despite advancements in technical approaches, increasing focus has shifted toward therapeutic interventions targeting the injurious pathways in ICH pathophysiology. Given that there is no approved therapeutic for ICH, intense research on ICH pathophysiology and disease course has been undertaken to identify suitable treatment targets [9,10].

Secondary injury following ICH and the development of perihematomal edema (PHE) drive further damage to the injured brain, which results in worsened neurologic deterioration; these processes evolve over hours to days post-ictus [10,11]. Therapies that interdict these processes would limit or even reverse further brain injury and would potentially benefit a wider array of patients. This rationale has endorsed intense research on edema and the immune response following stroke. The genesis and progression of PHE are intimately linked with the acute neuroinflammatory response to ICH. Indeed, the progression of PHE is closely correlated with the active processes of the inflammatory cascade unleashed by ICH [10]. Featured in these pathological pathways are the interactions between immune cells, intrinsic brain cells, and the cellular constituents of the neurovascular unit [12]. In terms of immune cells, the actions and implications of hematogenous monocyte/macrophages and neutrophils in the hemorrhagic brain have been the focus of the study; however, recent studies have identified the role of T lymphocytes to be active participants in the early immune response to ICH as well as contributors to PHE [13]. Notably, a proof-of-concept study modulating T cell response in ICH patients has demonstrated their involvement in secondary injury and outcome and has subsequently informed ongoing clinical trials targeting T cells in ICH [14,15]. In appreciation of growing experimental knowledge, this review compiles experimental and clinical findings describing the interplay between T cells and the NVU in ICH, as well as extrapolating known T cell interactions with the cells that form the NVU. In compiling evidence of these less-known interactions in ICH and interpreting the consequences of T cell/NVU actions, we aim to highlight their roles in contributing to secondary ICH injury and raise further questions remaining. Considering the involvement of the dysregulated NVU in the progression of cerebral edema, characterizing their interactions with T lymphocyte responses fills in the color towards a more complete understanding of the pathophysiological processes of secondary ICH injury and recovery, which may yield valuable insights towards new research areas in ICH pathophysiology.

## 2. NVU in ICH Pathophysiology

The extensive metabolic need of the brain is sustained by an extensive vascular network that constantly provides the necessary nutrients and substrates, removes waste and by-products, and regulates the entry of peripheral components into the parenchyma [12]. These vital functions are coordinated by the pervasive interaction and contact between the cells of the cerebrovasculature and those of the parenchyma. The term “neurovascular unit” has been adopted to reflect the close functional connection between brain cells and the microvasculature. Given this conceptual framework, the NVU is the assemblage of neurons, glia, pericytes, the extracellular matrix, and endothelial cells, which coherently functions to maintain brain homeostasis, regulate cerebral blood flow, and respond to stimuli. As each NVU component plays an active and specialized role in maintaining the reciprocal, dynamic linkages under physiological conditions, perturbations of individual elements consequently affect the NVU as a whole. Importantly, the NVU is a critical structural and functional element of the BBB; ICH degradation to the BBB involves damage to the NVU and its components [16].

The NVU and its dysfunction are prominently represented in the pathophysiology of ICH secondary injury. During hemorrhage, the mechanical forces generated in hematoma formation kill and damage tissue at the injury site and adjacent regions. In response to injury, microglia and astrocytes are rapidly activated and trigger the neuroinflammatory cascade [16]. Simultaneously, injured neurons, endothelial cells, pericytes, and astrocytes release a variety of signaling molecules (DAMPs, PAMPs, etc.), which stimulate peripheral leukocytes to home to the hemorrhagic brain. The concurrent accumulation of proteins, reactive oxygen species, cytokines, and chemokines, along with the iron and hemoglobin from the breakdown of erythrocytes, engenders an oxidative and inflammatory environment within the brain [3,10]. As a result, BBB disruption and NVU dysfunction occur, leading to cerebral blood flow reduction, progressive brain edema, and altered homeostasis, are key contributing factors to poor ICH outcomes [9].

## 3. T-Lymphocytes in the Immune Response to ICH

Immunity is an omnipotent factor throughout the continuum of ICH pathophysiology; the dynamics of immune response to ICH influence the course of the disease and ultimately inform patients’ outcomes. ICH swiftly elicits an immune response that extends beyond the CNS to include peripheral immune compartments [17,18]; ICH’s impact on peripheral immune organs is well illustrated in the atrophy of the spleen by the third-day post-injury, visualized by MRI imaging [18]. The post-ICH inflammatory response is mediated in part by cellular components; within the brain, microglia are the first immune responders to injury and initiate the inflammatory cascade [19,20]. Within hours after injury, peripheral leukocytes detect signals emanating from the injured brain, are activated and begin migrating to the brain. Infiltrating neutrophils and monocytes are reciprocally influenced by the local inflammatory environment, where they can amplify acute inflammation as well as initiate repair processes by participating in hematoma resolution [16]. In a clinical setting, peripheral leukocyte count has been associated with an increased risk of early neurological deterioration, increased long-term mortality, and poor functional outcomes [21]. Research on the immune response following hemorrhagic stroke initially focused upon the actions of the innate immune response; however, recent research increasingly registers that the cellular components of the adaptive immune wing in the acute ICH immune milieu.

Within the CNS, T cell presence is generally considered pathogenic; however, recent studies have found T cells patrolling the healthy cerebrospinal fluid to maintain immune surveillance of CNS borders suggesting T cell presence and function within the healthy CNS [22]. T lymphocytes are cells of the immune system with central functions in adaptive immunity. T cells are formed in the bone marrow by hematopoietic stem cells (HSC); HSC-derived developing T cells migrate to the thymic cortex to undergo maturation in an antigen-free environment [23]. The small proportion of T cells reaching maturity by surviving the selection process in the thymus further differentiate into T cell subtypes that play vital roles in controlling and shaping the immune response [23]. Two major T cell subtypes, CD4+ “Helper” T cells and CD8+ “Cytotoxic” T cells, work in conjunction in a highly coordinated process to mediate immune-mediated cell death. Reductively, CD4+ T cells are needed for activation of other cells, such as B cells and macrophages, and cytotoxic T cells kill harmful or damaged cells. Other prominent T cell subtypes include memory T cells, which remember previously encountered antigens, and T-regulatory cells, which moderate the immune response of other leukocytes. Within these major groups, yet more T cell subtypes have been described, such as Th17 cells or unique T cell receptor-bearing γδ T cells; differentiation towards these subtypes may be elicited under specific conditions of injury or disease [24].

**Figure 1:**
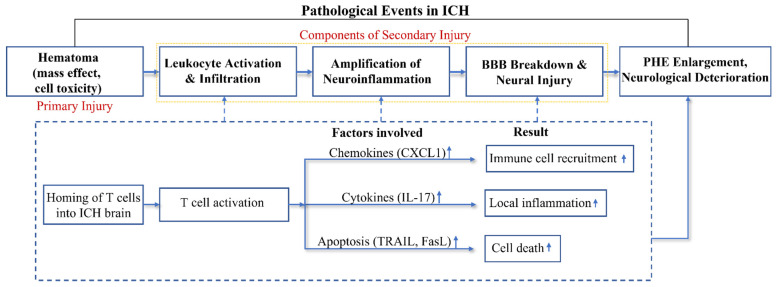
**Acute T cell actions in ICH.** After onset of ICH (hours to days), CD4+ T cells migrate to the brain, where they operate through antigen-dependent and/or antigen-independent mechanisms. The infiltrating CD4+ T cells may harm brain tissue through the release of pro-inflammatory cytokines (IL-17) and the promotion of local inflammation (a). They also secrete chemokines such as CXCL1 that can recruit circulating neutrophils into PHE (b). Their expression of the cell apoptosis ligands can induce apoptosis of intrinsic brain cells that express cognate apoptotic receptors (c). Such events (a–c) enhance neurovascular inflammation and exacerbate BBB disruption, leading to PHE expansion and neurological deterioration.

Recently, the consequences of T cell dynamics in the acute phase of ICH are increasingly appreciated. Experimental models of ICH revealed that of all systemic leukocyte populations, CD4+ T lymphocytes were the predominant populations, in both the well-established collagenase and autologous blood murine ICH models [25], at 24 h post-induction and persisting up to 14 days thereafter. This provocative finding has since been reinforced by our own work and those of others [13,26]. The abbreviated period between ICH induction and subsequent T cell infiltration suggests their antigen-independent actions in the injured brain since the generation of a systemic antigen-specific immune response is unlikely within a 24-h time span. Notably, infiltration of CD4+ T cells drastically outnumber CD8+ T cells in the ICH brain, and these CD4+ cells primarily aggregate around the site of injury, coinciding with the PHE region [25]. Depletion experiments and studies limiting T cell infiltration into the brain indicate that general T cell presence in the acute phase of ICH injury is detrimental, impacting the inflammation, BBB coherence, and edema volume [13,27]. Although a detailed mechanistic understanding and to what degree infiltrating T cells are associated with the detrimental effects remain open, their involvement in the processes of secondary injury and ICH pathophysiology is substantiated by clinical and experimental studies (Figure 1). The simultaneously harmful and protective immune processes accentuate the complexity of the ICH immunity; as a potentially modifiable determinant of ICH outcome, understanding of T cell action in ICH pathophysiology is necessary for the translation of neuroprotective strategies for this disease.

## 4. T Cell Crosstalk with Cells of the NVU

The crosstalk between T cells and cells in the ICH brain has reverberating implications for the NVU. The exact mechanisms of T-cell mediated injury to the NVU are currently unclear; however, an array of cytokines released by T cells are known to promote the harmful inflammatory environment of the ICH brain. Early infiltrating T cells to the ICH brain exert their immunomodulatory actions by releasing a collection of cytokine and chemokines; the cytokine array of T cells in the ICH brain at day 3 catalog an increased relative expression of IL-17, CXCL1, IFN-y, MMP9, IL-12 and IL-23 [26,28]. Within the stroke brain, CD4+ T cells are the major source of the pro-inflammatory cytokines IL-17 and IFN-y [26,28]. IL-17 is well known to prime neutrophilic inflammation, but its effects also extend to numerous cells that form the NVU. There is evidence that IL-17 can affect the vascular wall since human endothelial cells and pericytes strongly express the cognate receptor, IL-17RC; culture experiments of human pericytes exposed to IL-17 stimulate their activation and production of inflammatory molecules IL-6 and IL-8 [29]. Pericytes activated by IL-17 are shown to polarize neutrophils towards a pro-inflammatory phenotype, which subsequently synthesizes IL-1α, IL-1β, TNF, and IL-8, as well as prolonging neutrophil lifespan [29]. Astrocytes, a major component that maintains NVU structure, are also reactive to major T cell-released cytokines IL-17, IGN-g, TNF-a, and IL1b [30]. Astrocytes can be activated by any of these cytokines, which translocate its transcription factor NF-kB to shift towards a pro-inflammatory state where they further promote inflammation in ICH [20,30]. Importantly, T cells interact with microglia to enhance their activity under inflammatory CNS conditions with resounding consequences on NVU function. Clinical and experimental studies show how subsets of brain infiltrating T cells can influence microglial polarity in the acute phase of ICH [31,32]. Activated CD4+ T cells, as well as infiltrating γδ T cells, prime microglia and hematogenous macrophages via the IL-17/IL-23 axis to enhance their pro-inflammatory actions, which ultimately worsens hemorrhagic injury [32]. Conversely, T cell interactions with microglia may also confer beneficial responses; adoptive transfer of Treg (CD4+ CD25+FoxP3+) cells in the autologous blood ICH model has the ability to attenuate cerebral inflammation by suppressing the actions of classically activated microglia, this resulted in improved BBB stability, reduced cerebral edema, and alleviated cell death [33]; Treg restraint of microglial NF-kB and p65 was also shown in vivo [34]. The relevance of these findings in humans is speculated in the detection of the increased proportion of activated T cells and Tregs in the peripheral blood of patients at day three post-hemorrhage [33,34]. In all, these studies reveal the extensive interactions between acutely infiltrating T lymphocytes across the cellular composition of the NVU; these studies report the functional consequences of how T cells influence individual cell types to enact detrimental effects upon the NVU, the full characterization of how T cells modulate NVU functions as a unit requires further investigations. Moreover, although experimental studies have elucidated both beneficial and harmful effects on the NVU and the ICH brain, the predominance of activated CD4+ cells at the early timepoint intimates their general effect in early ICH injury. The identification of T lymphocyte subsets which polarize microglia and hematogenous macrophages towards reparative actions, even in the early phase, could present a potential endogenous pathway that may be amplified through therapeutic strategies. Little is currently known about T cell actions in the chronic phase of ICH; however, studies in acute ischemic stroke pathophysiology report their increased protection through differential subtype presence [35,36]. Nevertheless, these studies affirm the key effector function of T cell interactions with NVU cells and their resounding influence on secondary ICH injury and outcome.

## 5. T-Cell-Mediated Endothelial and NVU Dysfunction

In our current knowledge, the interactions between T-lymphocytes and cerebral endothelial cells following hemorrhagic strokes centers upon the endothelium, these interactions have progressive consequences in terms of aberrant NVU function and, ultimately pathophysiology. Post-stroke endothelial damage and dysfunction simultaneously affect the local inflammatory environment through augmentation of inflammatory processes and oxidative stress [37]; damage to the blood–brain barrier results in its diminished integrity and functional capacity, and thrombo-vascular complications may affect vascular tone and cause secondary hemorrhages [38,39].

The resulting endothelial cell dysfunction involves the production of reactive oxygen metabolites, swelling, or detachment from the underlying basement membrane, and compromised barrier function leading to increased protein extravasation and interstitial edema [40,41]. These events generally occur in post-capillary segments of the microvasculature and are often accompanied by the adhesion of leukocytes. T-cell orchestrated damage to the endothelium is vividly depicted in a mouse model of ICH in which triple staining of CD31+ endothelial cells, CD3+ T cells, and tight junction proteins at acute time points following model induction [15]. Zhang et al. visualized through immunofluorescent staining of ICH brain slices; confocal imaging revealed a close co-localization of CD3+ T cells around the blood vessels marked by CD31+ endothelial cells [15]. The presence of these infiltrating T lymphocytes further disrupted BBB architecture through the reduced expression of cerebrovascular tight junction proteins; tight junctions (TJ) are a type of connection between two endothelial cells and can limit the transfer of water and other substances through the BBB [42,43]. Inhibition of T lymphocytes following the ICH model, through genetic mouse lines or pharmacological blocking, rescued the expression of major TJ proteins and improved the BBB integrity, reflected by the reduced leakage of fluorescent markers, in the acute time points post-ICH. The functional consequences of limiting T cell entry into the hemorrhagic brain are experimentally shown in the reduction in brain water content in the first week post-ICH as well as the improvement of functional outcomes of ICH mice, measured by a battery of motor-sensory tests [13,15].

These findings indicate that infiltrating T cells may interact with the cerebrovasculature in the acute phase after ICH to aggravate damage to the BBB, which results in increased brain water volume and worsened outcomes in experimental models. Th17 and CD8+ T cells can cause endothelial dysfunction or death following ICH [26,32,42]; however, genomic sequencing data identifying other antigen-dependent and independent mechanisms suggest the more complex action of T cells, which remain to be determined in the ICH brain [44]. With the adoption of single-cell RNA sequencing, we now have the ability to catalog the transcriptomic profile of T cells at an individual resolution across time and specific compartments, giving insights into the mechanisms underlying their damage to the endothelium. Recent reports leveraging single-cell sequencing of blood and hematomal blood samples have elucidated possible pathways and the implications of T cell–endothelial interactions in ICH. With this approach, Durocher et al. sampled the peripheral blood of 18 ICH patients, with the average blood draw at approximately 60 h post-ictus, to determine the transcriptional changes of circulating leukocytes and further correlate enriched signaling pathways with clinical endpoints of total ICH volume and PHE volumes [44]. Through per-gene analysis for association with volumetric parameters (ICH & PHE volumes) paired with weighted gene co-expression network construction (WGCNA), study authors report a positive correlation between ICH and PHE volumes with the transcriptome of peripheral T cells following ICH. T lymphocyte clustered WGCNA analysis detected 33 significant pathways positively associated with enlargement of relative PHE volumes; though most of these pathways were related to inflammatory processes, the calcium-induced T lymphocyte apoptosis pathway points to direct implications on endothelial and NVU impairment [44,45]. In our own work, single-cell sequencing of brain infiltrating T lymphocyte populations derived from an autologous blood injection mouse model, we observed an increase in apoptosis-related genes, Tnfsf10, Tnfrsf10b, and FasL at day 3 post-stroke [26]. These genes encode tumor necrosis factor-related apoptosis-inducing ligand (TRAIL) and Fas-Ligand, which induce apoptosis upon interactions with their cognate receptors. Flow cytometry revealed expression of TRAIL cognate receptor, DR5, was significantly upregulated on endothelial cells in the ICH brain; treatment with anti-DR5 monoclonal antibodies subsequently reduced endothelial cell death in experimental ICH [26]. Direct endothelial cell death caused by interaction with T cells is further supported in clinically derived data, wherein hematomal blood was sequenced and found enrichment of TNF-related apoptosis, which corresponds with the experimental findings [45].

Recent works highlight how leukocyte–endothelial interactions in the brain can affect vascular tone and cerebral blood flow through capillary stalling, the transient disruption of the microcirculation [46]. In experimental cerebral hypoxia, leukocyte adhesion to endothelial cells degraded the cerebral endothelial glycocalyx and increased expression of leukocyte adhesion molecules, VCAM-1 and ICAM-1, contributes to the disruption of the microcirculation [47]. Although this work was carried out in a stroke model, the authors speculate that cerebral inflammation and pericyte death, which are shared features across stroke subtypes, are the central factors influencing capillary stalling [48]. Still, the pathological consequences of capillary stalling and the disruption of the microcirculation remain to be clarified in ICH, and the extent of T cell contribution to this phenomenon is unclear.

Taken together, these findings provide evidence that T cell interactions with cerebral endothelial cells can result in endothelial dysfunction and death through direct contact [26]. Moreover, T cell presence in the injured brain disrupted the structural coherence of the BBB by reducing the web of TJ proteins that tether the endothelial cells together; ablation of T cells prior to experimental ICH rescued the expression of major TJs, reduced the leakage of tracking dye across the BBB, and notably decreased brain water volume post-ICH. It is well known that CD4 T cells have a broad effector capacity in directly the inflammatory response; their release of pro-inflammatory cytokines such as IL-17, MMP7, and IFN-y aggravates endothelial dysfunction, which may contribute to increased vessel permeability and vascular inflammation.

## 6. T Lymphocyte Trafficking to the Injured Brain

T cells rapidly sense injury and move to the hemorrhagic brain, where they accumulate in the regions surrounding the hematoma; T lymphocytes, primarily CD4+ cells, arrive at the perihematomal regions as early as 12 h following stroke [26]. The trafficking of these lymphocytes has broad implications on the local inflammatory environment within the ICH brain parenchyma; however, T cell migration into the injured CNS also has reciprocal implications on the vasculature and other homing leukocytes. Unlike neutrophils, which flow freely in the circulation, in their quiescent state, lymphocytes traffic throughout the periphery by continuous low-avidity tethering on the vascular wall [40]. A variety of integrins tethers peripheral lymphocytes to the counter-structures upon the endothelial, allowing for rolling and immune surveillance. Major lymphocyte-endothelial integrins include lymphocyte function antigen-1 (LFA1), intercellular adhesion molecule-1 (ICAM-1), vascular cell adhesion molecule-1 (VCAM-1), and very late antigen-4 (VLA-1). Indeed, preclinical studies have shown the increased expression of β2-integrins on T cells in the acute phase following experimental ICH [49]. The early presence of T cells in the ICH brain is dependent upon the coordinated expression of these tethering proteins on both activated lymphocytes as well as the capillary endothelium; interdicting this process has been identified as a potential intervention strategy in restricting secondary brain injury in stroke.

Upon injury, signaling molecules released by the injured brain are detected by peripheral lymphocytes via their array of cytokine and chemokine receptors, inducing a rapid upregulation and conformational change of integrins, resulting in their transition from a low- to high-affinity/avidity state. From here, they rapidly infiltrate the CNS towards the site of injury by traversing the endothelial basement membrane into the perivascular space to finally accumulate in the regions proximal to the hematoma [25]. Several lines of evidence suggest that this process of lymphocyte migration into the brain can augment damage to cerebrovasculature. Preclinical and clinical experiments have consistently demonstrated that the absence or restriction of T lymphocytes entry into the ICH brain preserves neurovascular integrity and restricts edema burden following experimental ICH [14,15,43]. Although the exact mechanisms underlying this benefit are not fully characterized, disparate studies provide clues towards how T cells may degrade the endothelium during trafficking. This notion is well illustrated by the effects of T-cell-derived matrix metalloproteinases, endo-proteinases critical for extracellular matrix maintenance and remodeling, which degrade the ECM, compromise BBB integrity, and expedite leukocyte infiltration through the basement membranes [50].

## 7. Sphingosine-1-Phosphate Receptor Modulators

Considering the manifold ways in which T cells contribute to the processes of secondary injury following ICH, efforts in developing therapeutic interventions have focused on strategies for limiting the acute infiltration of T lymphocytes into the CNS. To this end, preclinical research has identified various approaches varying from ablation with monoclonal antibodies and methods of restricting T cell egress and trafficking.

Sphingosine-1-phosphate receptors (S1PRs) are a class of G-protein-coupled receptors that respond to a lipid signaling molecule sphingosine-1-phosphate. The S1P-S1PR axis is broadly involved in many physiological processes, including immune cell trafficking and angiogenesis. The diversity of their physiological function is mediated in part by five sub-receptors (S1PR1–S1PR5), which are differentially expressed across cell type and organ compartments and elicit particular responses under varying conditions of health and disease. S1PR1 has been implicated in the regulation of multiple immune responses; this receptor subtype is widely expressed on CNS intrinsic cells, vascular endothelial cells as well as lymphocytes. In the context of this review, S1PR2 is another pertinent receptor, given its reported distribution in the cerebrovasculature. The beneficial effects of S1PR modulation in experimental ICH were first reported in 2013 by Rolland and colleagues, which tested fingolimod administration in two established murine ICH models and one rat ICH model [13]. Fingolimod (FTY720) is an FDA-approved S1PR modulator used in the treatment of relapse-remitting multiple sclerosis, a neuro-autoimmune disease. Fingolimod non-selectively modulates S1PR1, 3, 4, and 5, but its action in restricting lymphocyte egress from lymph nodes is thought to be mediated by the downregulation of T cell S1PR1; Rolland et al. hypothesized that restricting acute T cell infiltration following ICH would ameliorate neuroinflammation and limit secondary injury. To this end, a dose of 1 mg/kg fingolimod was intraperitoneally delivered to the collagenase model of ICH one-hour post-induction; at 24- and 72-h post-ICH, a significant reduction in brain infiltrating lymphocytes was observed in the treatment groups versus control [13]. Interestingly, fingolimod treatment significantly reduced total leukocyte presence at both time points; although a decreasing trend in infiltrating monocytes and granulocytes was observed, quantification of these subtypes did not reach statistical significance. In this same study, fingolimod reportedly reduced CD3+ cell expression of ICAM-1 and protein levels of IFN-γ, IL-17, and ICAM-1 in the brain were also reduced in the treated group at 72 h [13]. Assessment of functional outcomes demonstrated that a single dose of fingolimod reduced brain water content at acute time points and a battery of cognitive and motor-sensory tests showed treatment improved outcomes in the short and long term [13]. This important work demonstrated, in the ICH brain, the unambiguous contribution of T cells towards the acute neuroinflammatory milieu as well as the close relation of the immune response with the progression of edema. Since the initial report of fingolimod’s effects in ICH, numerous other preclinical studies have reinforced the neuroprotective findings and have enriched the characterization of its protective effects [51,52].

To evaluate the clinical effects of fingolimod, a small pilot study was initiated in a two-arm study of 23 primary ICH patients receiving oral fingolimod within 72 h of symptom onset for three consecutive days. Compared to standard management, those receiving fingolimod had improved relative outcomes, quantitatively reflected by Glasgow Coma Scores and the National Institute of Health Stroke Scale, within the first two weeks of treatment. Additionally, a greater proportion of the fingolimod treated group achieved recovery of neurologic function compared to those receiving symptomatic management alone [14]. Importantly, experimental patients did not record adverse events in relation to the control group. Assessment of biological parameters revealed that the fingolimod regimen significantly reduced both absolute PHE volume and relative PHE volumes compared to control groups; moreover, circulating lymphocyte counts were transiently reduced, plasma ICAM1 and MMP9 levels were also decreased, and vascular permeability was protected with FTY720 [14]. This proof-of-concept work importantly demonstrates the benefits of fingolimod treatment in ICH patients, albeit in a small study sample.

The well-reported adverse cardiac effects limit its implementation in ICH patients, many of whom may have related co-morbidities. The off-target effects of fingolimod are associated with its non-selective modulation of S1PR3 [53]. These limitations have prompted the development of 2nd generation S1PR agonists, most notably, Siponimod and RP101075. Siponimod (BAF213) selectively binds to S1PR1 and S1PR5; experimental studies in aged ICH mice show similar protective effects as fingolimod [54]. Currently, a randomized and placebo-controlled trial of BAF312 is ongoing in patients with ICH to study its efficacy, safety, and tolerability (NCT03338998). RP101075 is a selective S1PR1 agonist with a superior cardiovascular safety profile. RP101075 reduced the number of brain-infiltrating immune cells, enhanced BBB integrity, and attenuated cell death after ICH resulting in the attenuation of neurological deficits and PHE [55].

## 8. Summary

**Figure 2:**
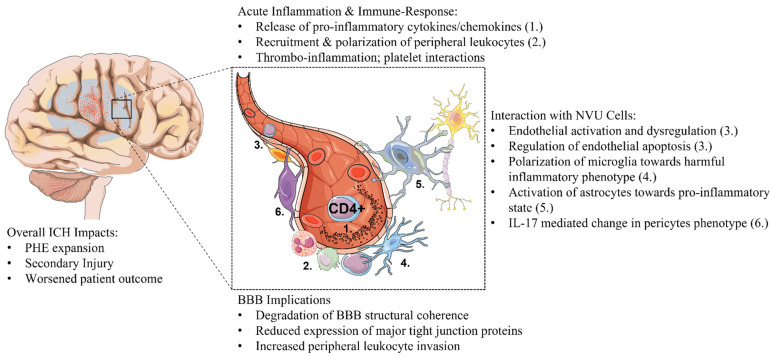
**T cell–NVU interactions in ICH**. Experimental studies show that infiltrating CD4+ T lymphocytes are rapidly activated and recruited to the hemorrhagic brain accumulating in the peri-hematomal regions. (a). Infiltrated T cells contribute to the inflammatory environment of the injured brain by releasing a battery of cytokines and are a factor in the recruitment of other peripheral leukocytes via chemokine release and endothelial interactions. (b). T cells interact with many NVU component cells; astrocytes, pericytes, and microglia can be activated and polarized towards a pro-inflammatory state. T cells can activate endothelial cells, cause their dysregulation, and directly cause endothelial apoptosis. (c). T cells damage the BBB by amplifying inflammation and reducing the expression of major tight junction proteins. The sum of these actions increases PHE and harmful inflammation to contribute to the progression of secondary ICH injury, which worsens outcomes.

Secondary injury following ICH is a modifiable aspect of the disease pathophysiology. The genesis and progression of perihematomal edema, blood–brain barrier deterioration, and infiltration of circulating immune cells are hallmark events in secondary ICH injury; the interaction between infiltrating immune cells and the neurovascular unit informs and intersects across these processes. In this context, the reciprocal cellular communication between T cells and cells of the NVU contributes to the interrelated mechanisms underlying PHE and inflammation in the ICH brain (Figure 2). T lymphocytes are the armamentarium of the immune system; they are highly susceptible to dynamic local and systemic cues that shape their activation status and differentiation towards sub-types. The major effector actions of activated T cells are increasingly implicated in pathophysiology and patient outcome following ICH. Clinical and experimental studies have revealed the significant contribution of T cell-NVU crosstalk in PHE and inflammatory pathways; studies targeting this interaction have importantly demonstrated functional improvements in patient outcomes and insights towards mechanisms of action. Current mechanistic knowledge of T lymphocyte-NVU action in ICH is confined to an understanding of how T cells interact with the distinct cellular components that form the NVU; since each component of NVU undertakes a unique role in the coherence of the unit, individual T cell-NVU cell interactions, therefore, possess reverberating ramifications in the hemorrhagic brain.

Activated CD4+ T cells shape the inflammatory environment of the ICH brain through the release of signaling molecules and direct interactions with NVU cells; through these actions, the NVU is dysregulated and leads to vasogenic edema and BBB degradation. CD4+ T cells are activated in the ICH brain and are major contributors to the cytokines IL-17 and IFN-y, which broadly act on infiltrating immune cells and brain intrinsic cells. IL-17 is a major T cell mediator of the innate immune response, particularly through inducing neutrophilic inflammation. In addition to CD4+ cells, Tregs in the acute phase also instruct neutrophil-directed BBB damage through PD-1, an important immune checkpoint receptor [56]. T cell promotion of early neutrophil action results in the physical blockade within the microvascular network, further reducing cerebral blood flow and direct entry into the brain parenchyma, followed by the release of granules containing enzymes and chemical species, which further injure brain tissue [57]. Similarly, T cell IL-17 signaling polarizes microglia and hematogenous macrophages, pericytes, endothelial cells, and astrocytes towards pro-inflammatory phenotypes, which collectively contribute to the detrimental inflammatory state of the ICH brain. T cells also direct post-stroke inflammation through the production of IFN-y, which contributes to the acute inflammatory and thrombogenic pathways, brain injury, and worsened neurologic deficits. Genetic knock-out model and antibody neutralization of major T cell cytokines alleviate BBB degradation, PHE volume, and neurovascular dysfunction to support T cell contribution to NVU-related pathogenic processes in acute ICH injury.

The crucial reciprocal interactions between T cells and endothelial cells influence the mechanisms of leukocyte trafficking and NVU-related processes in ICH pathophysiology. In the circulation, patrolling T cells adhere to the endothelium through the coordinated expression of cell adhesion molecules on endothelial cells. Upon their activation following stroke, the upregulated expression of major lymphocyte integrins (LFA-1, VCAM-1, VLA-1) integrins and increased avidity to their corresponding receptors on endothelial cells facilitate the rapid homing to the injured brain. IL-1b and TNF-a released by T cells prime the cerebrovascular endothelial cells to increase their expression of adhesion molecules to enhance peripheral leukocyte infiltration to the CNS. The course of T cell-endothelial adhesion and migration damages the NVU through T cell release of inflammatory MMPs and pro-inflammatory cytokines, which degrades the extracellular matrix and dysregulates cerebral endothelial cells. Moreover, ICH-activated T cell enrichment of TNFSF/TNFRSF regulates cellular apoptosis and directly induces cerebral-endothelial death through the TRAIL-DR5 and FasL-Fas axes. Interventional approaches that target these manifold pathways restrict edema volume, improve BBB coherence, and dampen neuroinflammation. The concerted actions of T cell-endothelial interactions are associated with the mechanisms of PHE enlargement, reduction in BBB integrity, and aggravation of secondary ICH injury.

## 9. Future Directions

Increasing evidence suggests a role for the migration of peripheral T cells into the brain after ICH. In the acute period, T lymphocyte interactions with the NVU are involved in the processes contributing toward the breakdown of the BBB, neuroinflammatory response, and PHE progression. The accumulated effect of T cell-NVU actions may have implications in secondary injury following ICH; patient-based and experimental findings show that strategies interdicting this interaction are neuroprotective and warrants further research. So far, evidence has shown that restricting the acute presence of these cells in the hemorrhagic brain is protective in a sample of ICH patients; further trials investigating the efficacy and safety of this strategy are ongoing. Experimental studies have yielded insights into how T lymphocytes contribute to the acute neuroinflammatory response in the ICH brain and their direct actions in affecting the NVU; however, much more is unknown than known regarding T cell role across the ICH disease course. Histological data of human ICH brains, supported by animal experiments, indicate that CD4+ T cells are the preponderant population of infiltrating T lymphocytes; however, what is known about the differential actions of other T cell subtypes in ICH remains fragmented and poses unanswered questions [26,28]. Animal experiments identifying the acute infiltration of γδT cells and their harmful contribution to the local inflammatory environment reflect the complexity of the immune response to ICH [32], the proportion to which γδT cells and T cells, in general, occupy in the immune response to ICH arises when considering the constellation of inflammatory processes and pathways. Moreover, experimental findings reporting Treg actions in constraining inflammation and involvement in reparative processes yield potential opportunities to identify endogenous protective and repair pathways to therapeutically leverage [32,36].

Of course, as new research sheds new light on aspects of the CNS and immunity, further questions will arise and direct new lines of questions. The discovery of lymphatic vessels in the meninges and evidence that T lymphocytes from the CSF space are drained into deep cervical lymph nodes via lymphatic vessels poses new questions related to the recruitment, kinetics, and persistence of T cells in the CNS parenchyma [22,58]. This updated concept of CNS immune privilege may also relate to the controversy of TCR-dependent pathways in acute brain injuries such as ICH [59]. The narrow focus of this review covering T cell–NVU interactions in ICH pathophysiology highlights the varied T cell involvement in post-ICH immunity, T cell effects on the NVU, and their consequences in the entangled network pathways of secondary injury. Further investigations characterizing the specifics of T cell populations involved in injury and repair, as well as their timing, are requisite for advancing our understanding of ICH and its possible treatment.

## Data Availability

Not applicable.
